# Integrating genes and metabolites: unraveling mango's drought resilience mechanisms

**DOI:** 10.1186/s12870-024-04908-w

**Published:** 2024-03-23

**Authors:** Xianbin Hou, Yu Kong, Zheng Teng, Cuifeng Yang, Yufeng Li, Zhengjie Zhu

**Affiliations:** 1 Guangxi Key Laboratory of Biology for Mongo, Baise University, Baise, 533000 China; 2 College of Agriculture and Food Engineering, Baise University, Baise, 533000 China

**Keywords:** *Mangifera indica* L., Drought, WRKY transcription factor 3, Polyamine oxidase 4, Protein MEI2-like 1

## Abstract

**Background:**

Mango (*Mangifera indica* L.) faces escalating challenges from increasing drought stress due to erratic climate patterns, threatening yields, and quality. Understanding mango's drought response mechanisms is pivotal for resilience and food security.

**Results:**

Our RNA-seq analyses unveil 12,752 differentially expressed genes linked to stress signaling, hormone regulation, and osmotic adjustment. Weighted Gene Co-expression Network Analysis identified three essential genes—*WRKY transcription factor 3*, *polyamine oxidase 4*, and *protein MEI2-like 1*—as drought defense components. *WRKY3* having a role in stress signaling and defense validates its importance. *Polyamine oxidase 4*, vital in stress adaptation, enhances drought defense. *Protein MEI2-like 1*'s significance emerges, hinting at novel roles in stress responses. Metabolite profiling illuminated Mango’s metabolic responses to drought stress by presenting 990 differentially abundant metabolites, mainly related to amino acids, phenolic acids, and flavonoids, contributing to a deeper understanding of adaptation strategies. The integration between genes and metabolites provided valuable insights by revealing the correlation of *WRKY3*, *polyamine oxidase 4* and *MEI2-like 1* with amino acids, D-sphingnosine and 2,5-Dimethyl pyrazine.

**Conclusions:**

This study provides insights into mango's adaptive tactics, guiding future research for fortified crop resilience and sustainable agriculture. Harnessing key genes and metabolites holds promise for innovative strategies enhancing drought tolerance in mango cultivation, contributing to global food security efforts.

**Supplementary Information:**

The online version contains supplementary material available at 10.1186/s12870-024-04908-w.

## Background

Mango (*Mangifera indica* L.) is a prized tropical crop with an illustrious history of over 4000 years [15]. Belonging to the Mangifera genus, it finds its roots in southern Asia, where it reigns supreme, accounting for an astounding 50% of global production and boasting a remarkable diversity [32]. To harness the potential of mango cultivation in arid and semi-arid zones, enterprising farmers have embraced advanced agricultural strategies, such as refined irrigation management techniques, to optimize the yield and quality of commercial mango crops [14].

However, despite the tenacity of mango trees in adverse climates, cultivating this cherished fruit takes time and effort. Among the most pressing concerns is the impact of drought stress on mango trees, which poses a formidable threat to sustainable mango production [26]. As climate patterns continue to fluctuate, understanding the intricacies of mango drought stress becomes an essential endeavor for researchers and cultivators alike. Unraveling mango trees' molecular, physiological, and ecological responses to drought stress is not only of scientific interest but also holds the key to devising effective strategies for mitigating its adverse effects on this invaluable crop [3, 21]. Previous studies have shed light on the resilience of mango trees to diverse environmental conditions. For instance, research by Rodriguez‐Dominguez et al. [30] investigated the diversity and distribution of mango cultivars in southern Asia. It highlighted the role of multiple domestication events and hybridization in shaping the impressive array of cultivars [36]. Moreover, the previous work explored the intricacies of mango irrigation management, offering valuable insights into enhancing water use efficiency in subtropical regions [25].

Despite these contributions, a comprehensive understanding of mango trees' genetic and physiological responses to drought stress still needs to be discovered. This study aims to bridge this critical knowledge gap by employing state-of-the-art methodologies to unravel the underlying mechanisms governing the adaptive responses of mango trees to water scarcity. By building upon the existing research and delving deeper into the intricacies of mango drought stress, our findings will offer a robust foundation for devising targeted interventions to bolster the resilience of mango crops in water-limited environments.

Plants exhibit a remarkable capacity to undergo molecular, biochemical, and physiological alterations in response to environmental stress, as emphasized by previous research by [38]. Among the vital mechanisms governing these adaptive responses is the activation or induction of defensive genes and transcription factors (TFs) in the wake of environmental cues, leading to critical transcriptional reprogramming essential for successful adaptation [34]. The intricate interplay between plant hormones and several TF families significantly influences the expression of drought-responsive genes. Notably, WRKY genes, often induced by salicylic acid, wield regulatory control over the expression of defense-related genes [40]. Given the profound impact of these genetic and molecular factors on a plant's ability to withstand drought stress, it becomes paramount to unravel the transcriptomic changes specific to *M. indica* L. in response to such challenging conditions [26]. By delving into the transcriptomic landscape of *M. indica* L. under drought stress, this study aims to provide a comprehensive understanding of the molecular events scoring its adaptive response. Unraveling the intricate web of gene regulation and transcriptional reprogramming in drought-stressed mango plants can offer invaluable insights into the underlying mechanisms that underpin drought tolerance. Furthermore, by identifying key TFs and defense-related genes that orchestrate this response, we lay the groundwork for future biotechnological interventions to enhance the drought resilience of this tropical crop [26].

Drought stress elicits profound changes in the metabolome of plants, representing a dynamic response to challenging environmental conditions [18]. The alterations in the metabolome serve as vital indicators of the plant's physiological state and provide crucial insights into the underlying mechanisms of drought tolerance [26]. One of the primary metabolic responses to drought stress involves the accumulation of compatible solutes or osmolytes, such as sugars (e.g., sucrose, glucose, and fructose), amino acids (e.g., proline), and polyols (e.g., sorbitol). These osmolytes act as osmoprotectants, assisting the plant in maintaining cellular water potential and preventing dehydration-induced damage to cellular structures and enzymes [31]. Additionally, compatible solutes can scavenge reactive oxygen species (ROS) generated during drought stress, thereby mitigating oxidative stress and preventing cellular damage [11]. Moreover, drought stress induces changes in primary metabolism, impacting carbon and nitrogen metabolism pathways. This metabolic reprogramming aids in reallocating resources to cope with the stress and ensure the plant's survival [24]. For instance, drought stress can lead to a shift from growth-related processes to resource-saving mechanisms, such as the accumulation of storage compounds like starch. Overall, metabolomics in the context of drought stress offers an essential dimension in comprehending the adaptive mechanisms of plants, fostering sustainable agriculture in the face of an uncertain climate future [23].

So, in this study, we sought to understand the molecular responses of three distinct mango cultivars (*M. indica* L.) to varying levels of drought stress. By subjecting the plants to low, moderate, and high drought stress conditions, we aimed to elucidate the intricate transcriptomic changes that occur in each cultivar under different degrees of water scarcity. We employed advanced techniques such as weighted gene coexpression network analysis to identify and characterize key drought-related genes within each cultivar. Through this ambitious approach, we aspired to shed light on the underlying mechanisms that govern the plants' adaptive responses to drought stress while revealing potential genetic factors contributing to drought tolerance. The insights gleaned from this study could potentially revolutionize our understanding of mango drought resilience. They may pave the way for developing climate-resilient cultivars, ensuring sustainable mango production in the face of an increasingly challenging climate.

## Methods

### Plant material and drought treatment

Three *Mangifera indica* L. cultivars were used in this study: ‘Tainong No.1 Mangan', ‘Jinhuang Mangan’ and ‘Guiqi Mangan’. Base Tiandong National Mango Germplasm Resource Nursery, China provided plant materials. No permission is required to work on this species. Voucher specimens are available in the GenBank Base Tiandong National Mango Germplasm Resource Nursery, China, under the numbers ASX671FT, ASX681FT, and ASX911FT, respectively. Prof Yufeng Li conducted official identification of the plant material. The experiment was conducted in a room temperature canopy, using two-year-old potted young trees with consistent growth as experimental materials. The experiment was performed between June and October, and mild, moderate, and severe drought treatments were applied on selected 20 plants per cultivar (mild drought soil moisture content of 50%; moderate drought soil moisture content of 40%; severe drought soil moisture content of 30%) and the control group (control soil moisture content of 60%) [39]. The water supply was supplied from 5 to 6 PM daily, and the amount of water replenished was controlled by weighing. The treatments applied are listed in Table 1.

**Table 1:** Treatments applied on three cultivars of *M. indica* L

Sample names	Treatment conditions
TC	Tainong Mango as the control group
JC	Jinhuang Mango as Control Group
GC	Guiqi Mango as the control group
TLD	Tainong Mango undergoes mild drought treatment
JLD	Jinhuang Mango undergoes mild drought treatment
GLD	Guiqi Mango undergoes mild drought treatment
TMD	Moderate drought treatment of Tainong Mango
JMD	Moderate drought treatment of jinhuang Mango
GMD	Moderate drought treatment of guiqi Mango
THD	Tainong Mango undergoes severe drought treatment
JHD	Jinhuang Mango undergoes severe drought treatment
GHD	Guiqi Mango undergoes severe drought treatment

After 20 days of drought stress, leaf samples were collected in three replicates per treatment and immediately flash-frozen in liquid nitrogen for further analysis.

### RNA extraction, library construction, and sequencing

According to the manual instructions, the ethanol precipitation protocol and CTAB-PBIOZOL reagent were used to purify total RNA from the plant tissue**.** A total amount of l ug RNA per sample was used as input material for the RNA sample preparations. Sequencing libraries were generated using NEBNextRUltraTMRNA Library Prep Kit for IlluminaR (NEB, USA) following manufacturer's recommendations, and index codes were added to attribute sequences to each sample. mRNA was purified from total RNA using poly-T oligo-attached magnetic beads. Fragmentation was performed using divalent cations under elevated temperature in NEBNext First Strand Synthesis Reaction Buffer(5X). The first strand of cDNA was synthesized using a random hexamer primer and M-MuLV Reverse Transcriptase (RNase H-). Second-strand cDNA synthesis was performed using DNA Polymerase l and RNase H. Remaining overhangs were converted into blunt ends via exonuclease/polymerase activities. After adenylation of 3' ends of DNA fragments, the NEBNext Adaptor with hairpin loop structure was ligated to prepare for hybridization. To select cDNA fragments of preferentially 250 ~ 300 bp in length, the library reagents were purified with the AMPure XP system (Beckman Coulter, Beverly, USA). Then 3 ul USER Enzyme (NEB, USA) was used with size-selected, adaptor-ligated cDNA at 37 °C for 15 min followed by 5 min at 95C before PCR. Then PCR was performed with Phusion High Fidelity DNA polymerase, Universal PCR primers, and Index (X)Primer.

At last, PCR products were purified (AMPure XPsystem) and library quality was assessed on the Agilent Bioanalyzer 2100 system. The clustering of the index-coded samples was performed on a cBot Cluster Generation System using TruSeq PE Cluster Kit v3-cBot-HS (Illumina) according to the manufacturer's instructions. After cluster generation, the library preparations were sequenced on the Illumina platform, and Campo generated 150 bp paired-end reads [27]. Clean reads were aligned against the reference genome [17]. FeatureCounts quantified gene expression levels to calculate the gene alignment and then calculate the FPKM of each gene based on the gene length.

### Gene differential expression and enrichment analysis

DESeq2 was used to analyze the differential expression between the two groups, and the *p-*value was corrected using the Benjamini & Hochberg method [29]. The corrected *p*-value and |log2foldchange| are used as the threshold for significant difference expression [35]. The enrichment analysis is performed based on the hypergeometric test. For KEGG, the hypergeometric distribution test is performed with the unit of the pathway; for GO, it is performed based on the GO term [2, 16, 37].

### Weighted gene co-expression network analysis

The gene co-expression network analysis was performed to identify defense-related hub genes in Mango against drought stress. This analysis was carried out using R programming (version 4.1.2), and default parameters were used [19]. The FPKM values were normalized, and an adjacency matrix was constructed. The adjacency matrix was converted into a topological overlap matrix (TOM) using the WGCNA package [4]. The transcripts with identical expression patterns were grouped into one module, and eigengenes were calculated for each module. Finally, the genes from each module were exported using default parameters to Cytoscape for network visualization [20].

### Metabolome analysis

#### Dry sample extraction

Using vacuum freeze-drying technology, place the biological samples in a lyophilizer (Scientz-100F), then grind (30 Hz, 1.5 min) the samples to powder form by using a grinder (MM 400, Retsch). Next, weigh 50 mg of sample powder using an electronic balance (MS105DΜ) and add 1200μL of -20 °C pre-cooled 70% methanolic aqueous internal standard extract (less than 50 mg added at the rate of 1200μL extractant per 50 mg sample). Vortex once every 30 min for 30 s, for 6 times. After centrifugation (rotation speed 12,000 rpm, 3 min), the supernatant was aspirated, and the sample was filtered through a microporous membrane (0.22 μm pore size) and stored in the injection vial for UPLC-MS/MS analysis [6].

#### UPLC Conditions

The sample extracts were analyzed using a UPLC-ESI–MS/MS system (UPLC, ExionLC™ AD, https://sciex.com.cn/; MS, Applied Biosystems 6500 Q TRAP, https://sciex.com.cn/). The analytical conditions were as follows: UPLC: column, Agilent SB-C18 (1.8 µm, 2.1 mm * 100 mm); The mobile phase consisted of solvent A, pure water with 0.1% formic acid, and solvent B, acetonitrile with 0.1% formic acid. Sample measurements were performed with a gradient program that employed the starting conditions of 95% A, 5% B. Within 9 min, a linear gradient to 5% A, 95% B was programmed, and a composition of 5% A, 95% B was kept for 1 min. Subsequently, a composition of 95% A and 5.0% B was adjusted within 1.1 min and maintained for 2.9 min. The flow velocity was 0.35 mL per minute; The column oven was set to 40 °C; The injection volume was two μL. The effluent was alternatively connected to an ESI-triple quadrupole-linear ion trap (QTRAP)-MS [12].

#### ESI-Q TRAP-MS/MS

The ESI source operation parameters were as follows: source temperature 500 °C; ion spray voltage (IS) 5500 V (positive ion mode)/-4500 V (negative ion mode); ion source gas I (GSI), gas II(GSII), curtain gas (CUR) was set at 50, 60, and 25 psi, respectively; the collision-activated dissociation (CAD) was high. QQQ scans were acquired as MRM experiments with collision gas (nitrogen) set to medium. DP (declustering potential) and CE (collision energy) for individual MRM transitions were done with further DP and CE optimization. A specific set of MRM transitions was monitored for each period according to the metabolites eluted within this period [8].

#### Principal component analysis, hierarchical cluster analysis, and pearson correlation coefficients

Principal component analysis (PCA) was performed by the statistics function prcomp within R (www.r-project.org). The hierarchical cluster analysis (HCA) results of samples and metabolites were presented as heatmaps with dendrograms. In contrast, Pearson correlation coefficients (PCC) between samples were calculated by the cor function in R and presented as only heatmaps. R package ComplexHeatmap carried out both HCA and PCC. For HCA, normalized signal intensities of metabolites (unit variance scaling) are visualized as a color spectrum [8].

#### Selection of differential metabolites and KEGG enrichment analysis

For two-group analysis, differential metabolites were determined by VIP (VIP > 1) and absolute Log2FC (|Log2FC|≥ 1.0). VIP values were extracted from the OPLS-DA results, which also contain score and permutation plots, and were generated using the R package MetaboAnalystR [9]. The data was log-transformed (log_2) and mean centering before OPLS-DA [33]. To avoid overfitting, a permutation test (200 permutations) was performed. Identified metabolites were annotated using the KEGG compound database (http://www.kegg.jp/kegg/compound/), and annotated metabolites were then mapped to the KEGG Pathway database (http://www.kegg.jp/kegg/pathway.html). Pathways with significantly regulated metabolites mapped were then fed into MSEA (metabolite sets enrichment analysis), and the hypergeometric test’s *p*-values determined their significance [16].

## Results

### RNA‑sequencing and the identification of candidate genes involved in Mangifera indica resistance against drought stress

#### Transcriptome profiling, differentially expressed genes, and enrichment analysis.

**Fig. 1:**
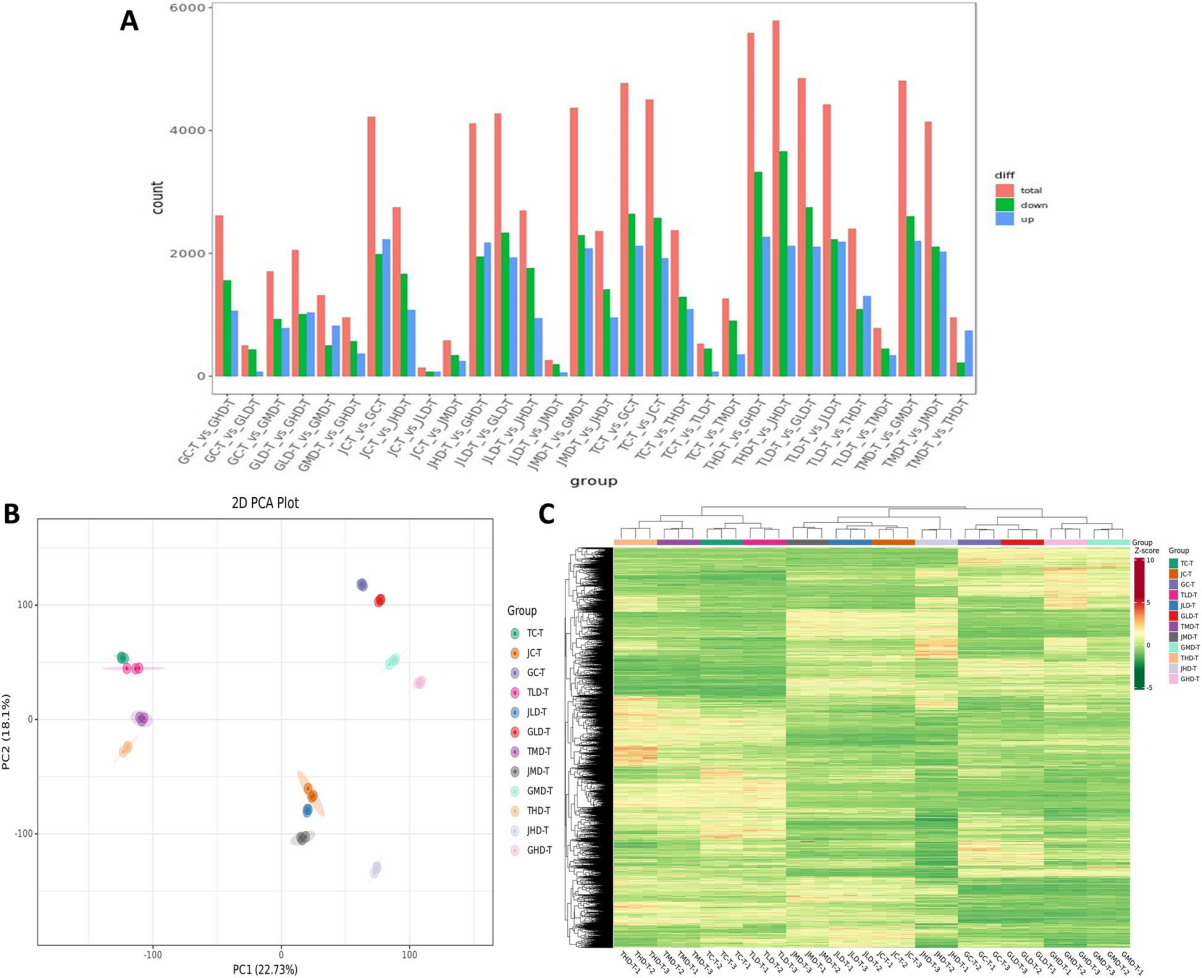
Transcriptome profiling of 3 M*. indica* L. varieties at light, moderate, and high levels of drought stress. **A** Differentially expressed genes (DEGs) in 30 possible combinations. **B** Principal component analysis (PCA) shows the clustering between different groups. **C** Hierarchical clustering (Heat map) showing up (red) and down (green) regulated genes

RNA-seq analysis of 36 mango samples from 3 different varieties under low, moderate, and high drought stress was performed through Illumina sequencing. Clean reads ranged from 42,431,360 to 52,498,358, and the Q30 base percentage ranged from 90.56% to 94.2% with a GC value of 42.09% (Supplementary Table 1). clean reads were aligned with the reference genome (GCA_011075055.1). Based on the comparison results, the gene expression analysis was performed, and a total of 12,752 differentially expressed genes were identified according to their expression levels | log2 (fold-change) |> 1 and an adjusted *p*-value < 0.05 in each pairwise comparison (Fig. 1A). The abundance of upregulated genes was lower than downregulated genes in all-pairwise comparisons except GLD vs GMD, JC vs GC, JHD vs GHD, TLD vs THD, and TMD *vs* THD. The principal component analysis (PCA) revealed the variability among transcriptome data of different samples. The clustering of samples of one group together and away from other and control groups indicates drought stress-induced changes in gene expression (Fig. 1B). A heatmap was constructed to visualize the expression pattern of DEGs in each comparison group, which showed high (RED) and low expression of DEGs (Fig. 1C).

DEGs were classified into different metabolic and cellular processes through the KEGG and GO enrichment analysis. The differentially expressed gene sets were allocated to significant KEGG pathways (*p* > 0.05), as illustrated in Supplementary Fig. 1. Among the paths enriched for resistance-related processes, the following emerged prominently: Phenylpropanoid biosynthesis, Plant hormones and signal transduction, ABC transporter, interactions with plant pathogens, glycolysis, Biosynthesis of amino acids, carbon metabolism, Circadian rhythm, starch, and sugar metabolism, as well as the MAPK signaling pathway. Similarly, in the GO enrichment analysis, a notable proportion of genes demonstrated significant associations with functions like Oxidoreductase activity, cellular anatomical entity, photosynthesis, lipid metabolic processes, and activities related to transmembrane transporters. (Supplementary Fig. 2).

#### Candidate gene identification through WGCNA

**Fig. 2:**
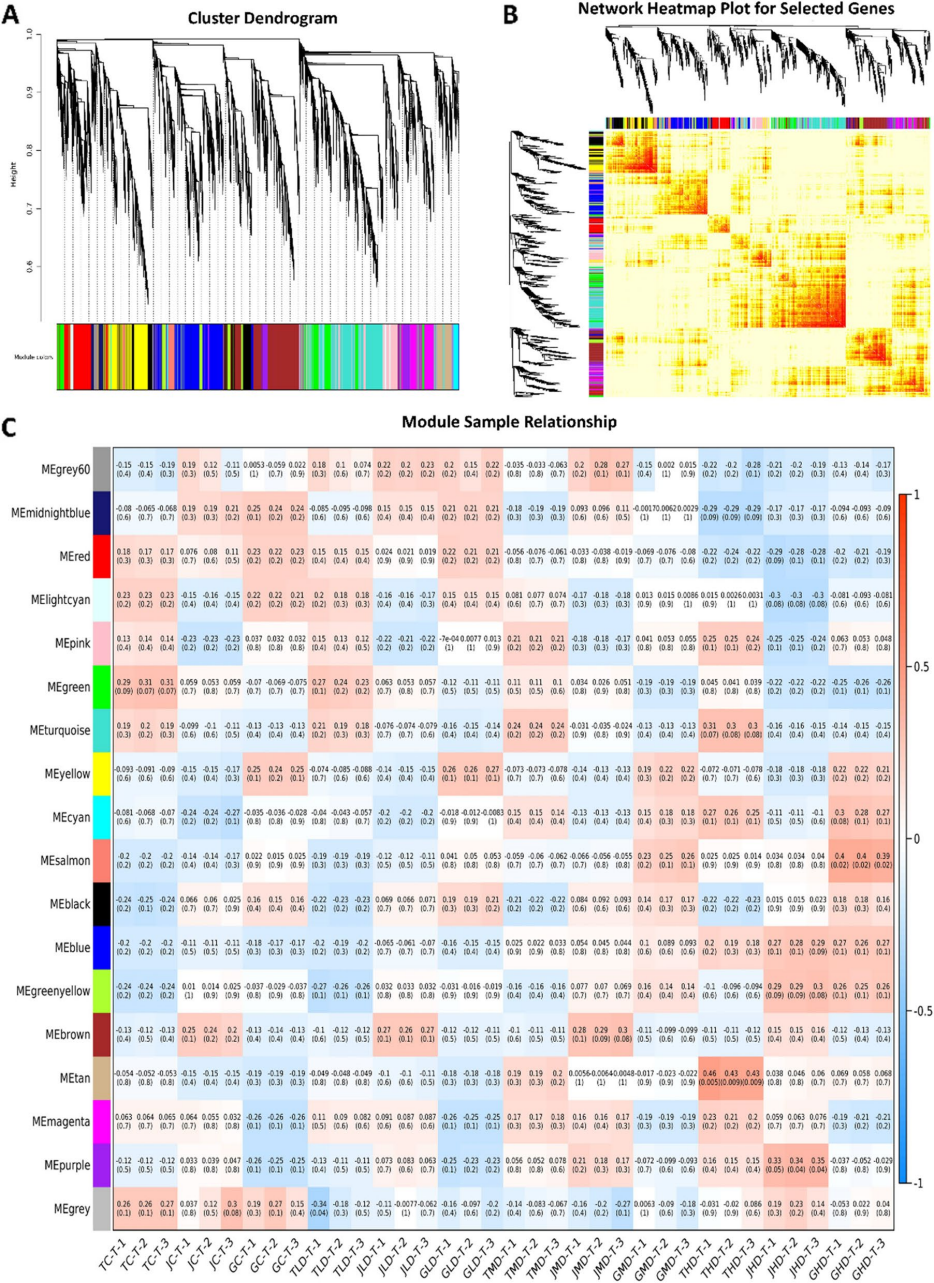
Weighted gene co-expression network analysis to identify essential candidate genes involved in mango resistance. **A** Cluster dendrogram of each sample. **B** Network heatmap of selected genes **C** Module-sample associations based on Pearson correlations. The color key from green to red represents r2 values ranging from–1 to 1

**Fig. 3:**
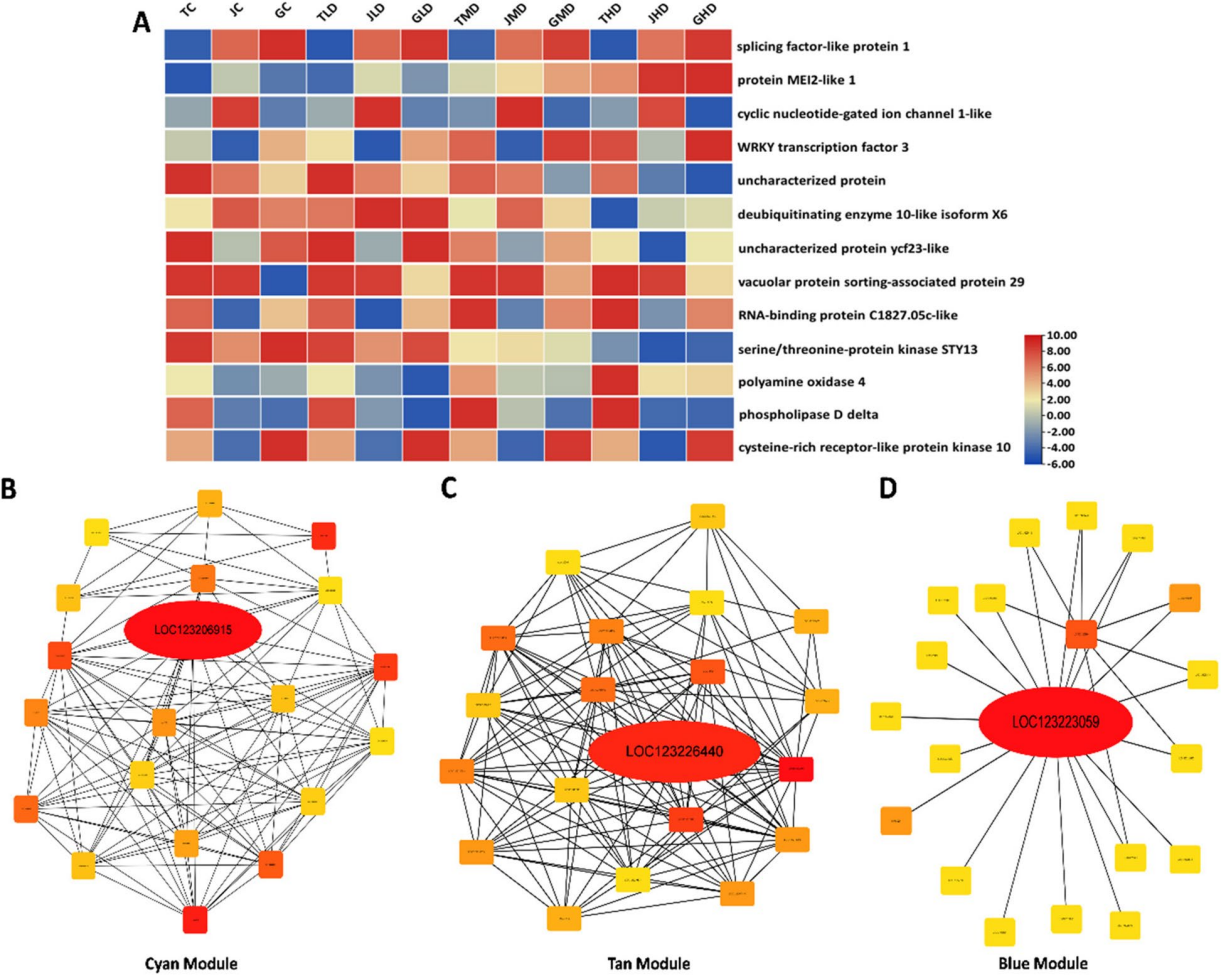
**A** Heat map showing the expression of 13 identified hub genes in 3 mango varieties under drought stress. Color bars indicate the expression of genes from low (blue color) to high (red color). **B**-**D** Gene networks of three highly correlated modules with critical genes involved in mango resistance to drought stress

The FPKM values of 12,752 DEGs were used for the weighted gene co-expression network analysis, and the module trait correlations were calculated. 17 gene modules were identified based on co-expression patterns. Each module is represented by a different color and presented as a cluster dendrogram and network heatmap (Fig. 2A and B). These 17 identified modules (Fig. 2C) include Black, Blue, Brown, Cyan, Green, Greenyellow, Grey60, Light cyan, Magenta, Midnight blue, Pink, Purple, Red, Salmon, Tan, Turquoise, and Yellow. The top 20 genes from each module were used to construct the networks to identify hub genes using the Cytoscape built-in extension, namely “CytoHubba,” to visualize the gene networks. Through network co-relation, one hub gene from 13 modules (total of 13 hub genes) was identified which include; LOC123205916 in the Black module, LOC123223059 in the Blue module, LOC123225476 in the Brown module, LOC123206915 in Cyan module, LOC123224172 in Green module, LOC123201570 in Grey60, LOC123219596 in Light cyan module, LOC123207090 in Magenta module, LOC123193440 in Pink module, LOC123221167 in Red module, LOC123226440 in Tan module, LOC123228963 in Turquoise module and LOC123219440 in Yellow module (Fig. 3A). Furthermore, gene expression analysis and gene annotation information were extracted from the reference genome to find out the key candidate genes involved in mango defense from these 13 hub genes within these gene networks. Finally, three key candidate genes involved in mango drought resistance mechanisms were identified. The identified vital candidates *are WRKY transcription factor 3* (LOC123206915) from the Cyan module, polyamine oxidase 4 (LOC123226440) from the Tan module, and protein MEI2-like 1 (LOC123223059) from the blue module (Fig. 3B-D).

#### Key candidate encoding defense mechanisms in M. indica against drought stress

**Fig. 4:**
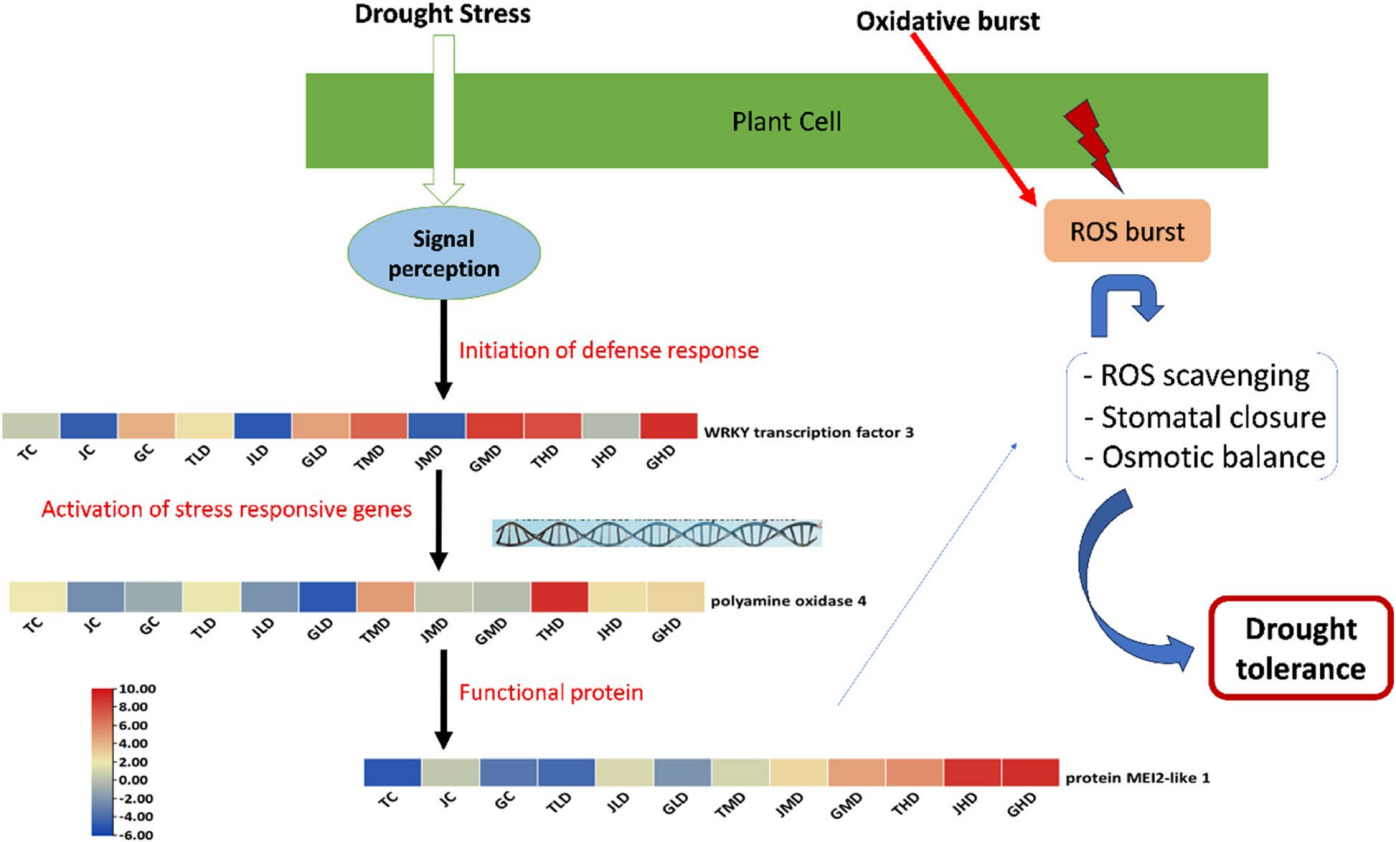
Schematic representation of the role of three key drought-resistant genes in inducing mango plant resistance against low, moderate, and high levels of stress

The three identified drought-resistant key genes in *M. indica* are identified as *WRKY transcription factor 3* (LOC123206915) from the Cyan module, *polyamine oxidase* 4 (LOC123226440) from the Tan module and protein *MEI2-like 1* (LOC123223059) from the Blue module. WRKY-3 transcription factor is involved in signaling pathways regulating stress-responsive gene expression. *WRKY-3* could regulate the expression of genes related to drought stress tolerance, such as those involved in stomatal closure, osmotic regulation, and ROS scavenging. They showed a consistent increase in expression with the increase in the level of drought stress from light to high in all three varieties. Still, the highest expression was observed in Guiqi (G), and the lowest was recorded in Jinhuang (J). *Polyamine oxidase 4* also showed a gradual increase in expression with the increase in the level of drought stress and the significantly higher increase was seen in Tainong (T) Mango variety at high level of drought stress as compared to its relative control. Polyamines are organic compounds that play diverse roles in plant growth and stress responses. *Polyamine oxidase 4* might be implicated in regulating the balance between polyamine levels and H_2_O_2_ production during drought stress. Similarly, gene *MEI2-like 1* was significantly increased in expression with the increase in drought stress levels in all three varieties compared to their relative controls. They were highly expressed in high drought-stressed conditions. The *MEI2-like 1* gene might be involved in post-transcriptional gene regulation, potentially impacting the plant's response to drought stress. RNA-binding proteins, like *MEI2-like 1*, can influence the stability and translation of mRNAs involved in stress responses. *MEI2-like 1* gene might be involved in post-transcriptional gene regulation, potentially impacting the plant's response to drought stress. RNA-binding proteins, like *MEI2-like 1*, can influence the stability and translation of mRNAs involved in stress responses. Through their consistent and significant expression and potential role in drought stress tolerance and plant defense mechanism, these three genes were screened as a drought resistant gene in *M. indica* plant (Fig. 4).

### Comparative metabolome analysis

#### Metabolome profiling and differentially expressed metabolites

**Fig. 5:**
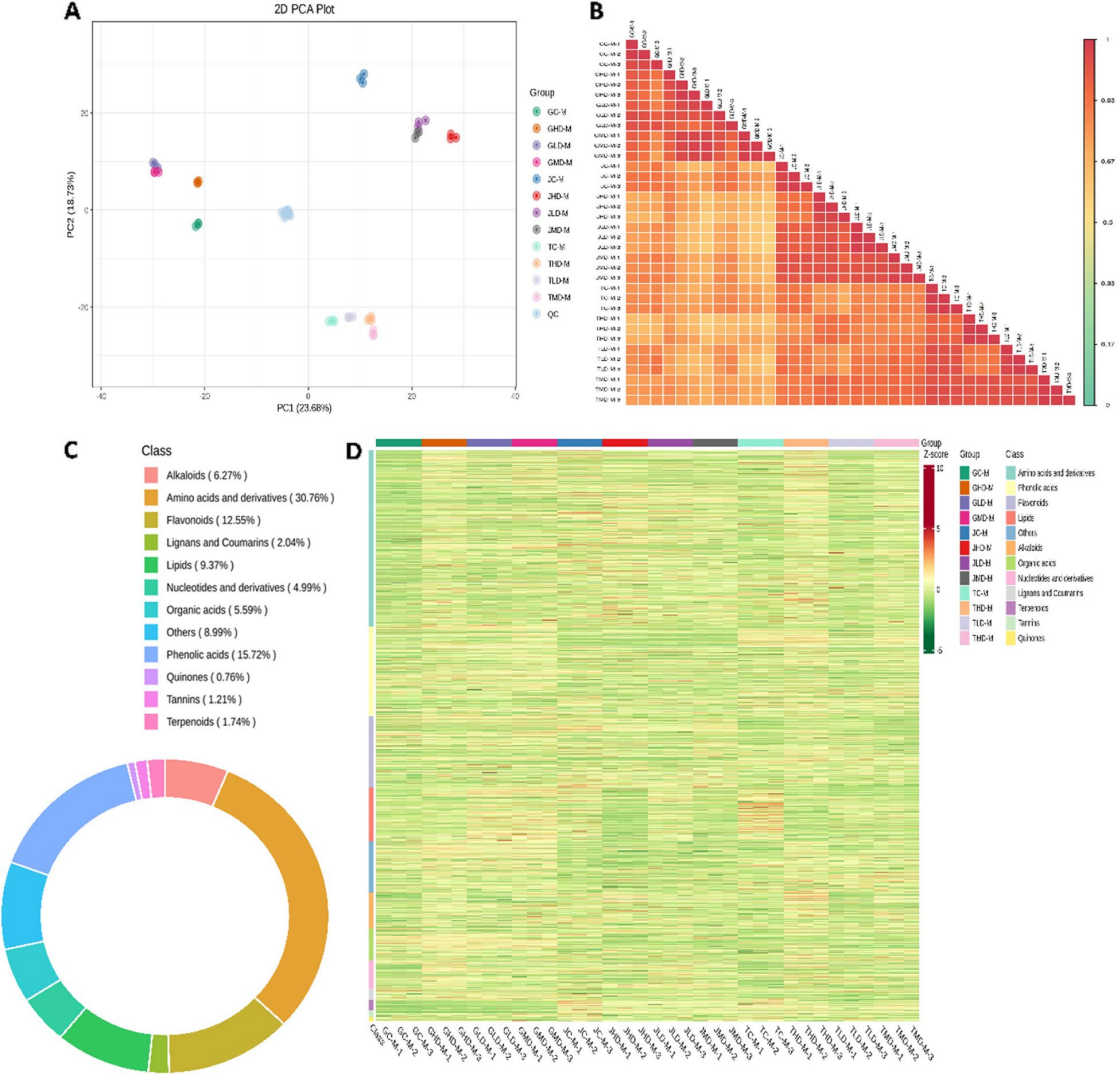
Metabolome profiling of three mango varieties under drought stress. **A** PCA plot shows a divergence in metabolome data among treatments. **B** Pearson correlation shows a positive correlation. **C** Pie chart showing the percentage distribution of differentially annotated metabolites in different classes. **D** Hierarchical clustering of identified abundant metabolites

To obtain complete insight into M. indica's response to drought stress of light, moderate, and high levels of drought stress in Guiqi (G) Jinhuang (J) and Tainong (T) varieties, we performed a widely targeted metabolome analysis of 36 samples through UPLC-ESI–MS/MS system. The principal component analysis (PCA) clearly showed the diversity in the samples. The clustering of samples of each group away from the samples of other groups showed the diversity among treatments and metabolomic data (Fig. 5A). Through Pearson correlation between different groups based on identified metabolites, the positive correlations indicate that the metabolite profiles tend to change similarly across different conditions. For example, a strong positive correlation exists between GC and JC (r = 0.96), meaning the identified metabolites in GC tend to increase/decrease similarly to those in JC. The same is true for GHD/JHD, GLD/JLD, etc., showing the correlated metabolic profiles across those conditions. The strength of the correlations (many above 0.9) suggests the metabolic changes are highly consistent across those paired conditions. This may indicate the conditions represent similar degrees of some treatment or intervention as we hypothesized earlier. The tight clustering on the correlation plot also shows that the relationships are linear. This matrix indicates that the identified metabolites are changing very correlatedly across the different conditions. The high positive r values suggest the metabolite profiles shift consistently across paired conditions like GC/JC or GHD/JHD (Fig. 5B).

One thousand three hundred and twenty-three significant metabolites were detected, from which 990 were differentially annotated (Supplementary Table 2), and abundant metabolic features were filtered based on different classes (Fig. 5C). The metabolites fall into a total of 12 classes, and the abundance of metabolites from each sample group in each of the 12 classes was represented through a heatmap (Fig. 5D). Amino acids and derivatives were the most abundant group, with 333 metabolites in this class. Phenolic acid was considered the second most abundant class with 162 metabolites. A total of 141 metabolites falls in the Flavonoid class. Lipids, alkaloids, organic acids, nucleotide derivatives, Lignans and coumarins, terpenoids, Tannins, Quinones, and others comprise 89, 53, 50, 47, 23, 20, 13, 4, and 55 metabolites (Supplementary Table 2).

#### KEGG annotation and enrichment analysis

According to the differential metabolite results, the KEGG pathway enrichment analysis was performed, where the Rich Factor is the ratio of the number of differential metabolites in the corresponding pathway to the total number of metabolites annotated by the pathway, and the higher the value, the greater the degree of enrichment. Using the KEGG annotation information of the differential metabolites identified according to the screening criteria, the top 20 significantly enriched KEGG metabolic pathways were selected, and all the differential metabolites in these pathways were clustered to better study the changes of the content of substances in potentially important metabolic pathways in different groups (Supplementary Fig. 3).

#### Expression pattern of key metabolites

**Fig. 6:**
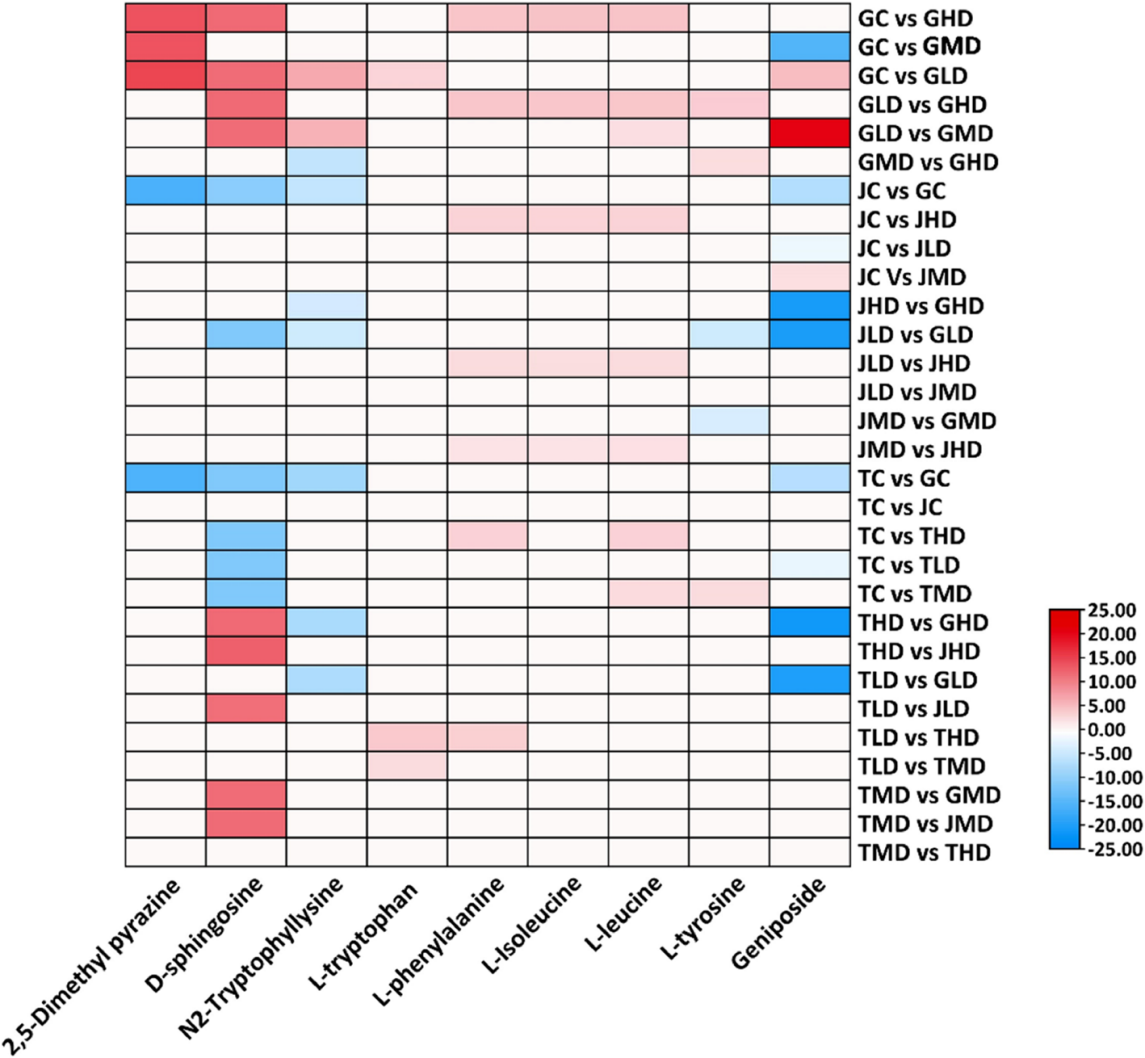
Heatmap showing the old change expression of top 9 key metabolites in 30 possible combinations of three mango varieties under low, moderate, and high levels of drought stress

The differences in the quantitative results of metabolites in different groups were calculated, and the top 9 metabolites with the largest absolute value of log_2_FC were selected based on the differential metabolites identified by the screening criteria (Fig. 5). The highly expressed metabolite in each group includes 2,5-Dimethyl pyrazine, D-sphingosine, N2-Tryptophyllysine, L-tryptophan L-phenylalanine, L-Isoleucine, 17-Hydroxylenolenic acid, L-leucine, L-tyrosine, Geniposide. Expression is shown in Fig. 6. In the heatmap analysis, notable changes in metabolite expression emerge across different sample comparisons. In the comparison between GC and GHD, several metabolites like 2,5-Dimethyl pyrazine, D-sphingosine, L-phenylalanine, L-Isoleucine, L-leucine, and L-tyrosine are upregulated, while Geniposide shows a downregulation. A similar trend is observed when GC is compared to GLD, where multiple metabolites are upregulated. In the comparison of GLD and GHD, there is an upregulation of D-sphingosine, L-phenylalanine, L-Isoleucine, L-leucine, and L-tyrosine. In GLD *vs* GMD, metabolites such as D-sphingosine, N2-Tryptophyllysine, and Geniposide are upregulated. In GMD *vs* GHD, L-tyrosine and Geniposide show higher expression. The JC *vs* GC comparison demonstrates the downregulation of several metabolites in JC. Conversely, JC *vs* JHD reveals upregulation of L-phenylalanine, L-Isoleucine, L-leucine, and L-tyrosine. L-phenylalanine exhibits downregulation in JC *vs* JLD. Meanwhile, JC *vs* JMD showcases an upregulation of L-tyrosine. These insights illuminate the dynamic changes in metabolite expression within diverse sample contexts.

### The interplay between key genes and metabolites

**Fig. 7:**
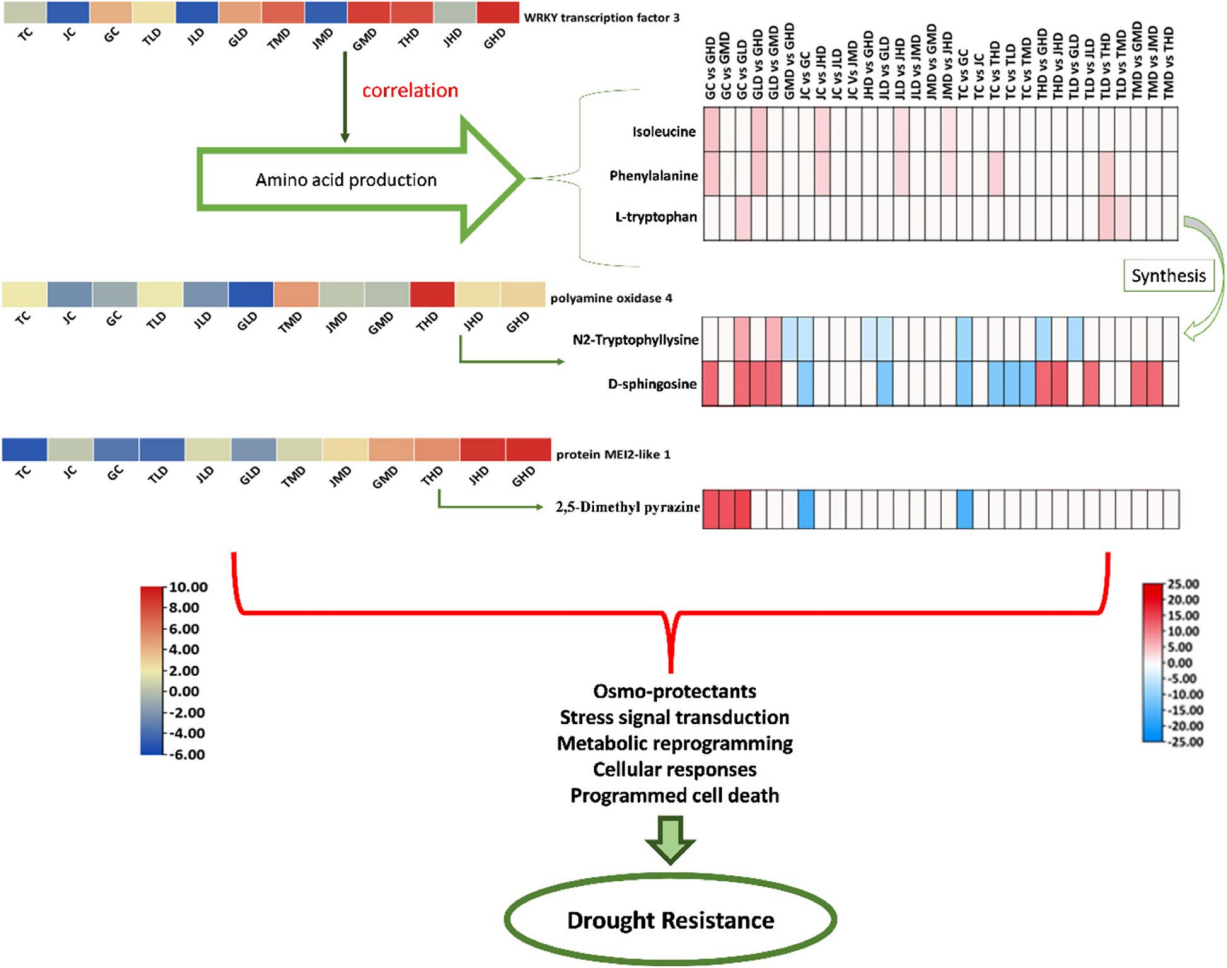
Schematic representation of the correlation between key genes and metabolite and their role in induced mango resistance to drought

The present investigation delves into the interplay between genetic determinants and metabolic dynamics within the framework of mango tree-responsive strategies to drought-induced stress conditions, thereby elucidating a description of sophisticated adaptive mechanisms. Among the pattern of pivotal genes is the WRKY transcription factor 3 (LOC123206915), which assumes a central role in this intricate process (Fig. 7). The engagement of this gene coincides with the heightened accumulation of specific amino acids, namely L-tryptophan, L-phenylalanine, and L-isoleucine. These amino acids, recognized not only for their roles as osmo-protective agents but also as pivotal participants in stress-responsive signaling cascades and metabolic recalibration, contribute to the multifaceted nature of the plant's drought coping strategies.

The significance of Polyamine oxidase 4 (LOC123226440) is equally compelling, establishing a symbiotic relationship with metabolites including D-sphingosine and N2-Tryptophyllysine (Fig. 7). The regulatory influence of D-sphingosine on stress-activated cellular responses, encompassing programmed cell death, finds resonance with the well-established role of polyamines in steering the plant's adaptation to drought-induced challenges. N2-Tryptophyllysine, potentially arising from L-tryptophan, emerges as a plausible modulator of protein functionality under arid conditions, thereby exerting a discernible impact on the plant's strategies for mitigating stress.

The intriguing relationship between the protein MEI2-like 1 (LOC123223059) and 2,5-Dimethyl pyrazine introduces complexity (Fig. 7). While the precise functional range of MEI2-like 1 remains an ongoing subject of exploration, the prominence of 2,5-Dimethyl pyrazine, recognized for its pivotal involvement in plant–microbe interactions and stress-related signal transduction pathways, furnishes valuable insights into the conceivable role of MEI2-like 1 in mediating stress-induced interplays.

## Discussion

Mango, a widely cultivated fruit crop, faces escalating challenges due to the increasing drought stress caused by erratic climatic patterns [26]. Drought stress substantially threatens mango cultivation, leading to yield losses and compromised fruit quality [38]. In response to these environmental constraints, plants orchestrate intricate molecular and physiological adaptations to mitigate the detrimental effects of water scarcity [38]. Unraveling the mechanisms underlying mango's response to drought stress is paramount for enhancing crop resilience and securing food production. Like other crops, Mango plants enact a series of physiological, biochemical, and molecular adjustments to combat the adverse impacts of drought stress [22, 32]. These responses are orchestrated at various levels to ensure survival and sustain growth under challenging conditions. Emerging genomic research has spotlighted the pivotal role of genes in orchestrating mango's defense against drought stress. RNA-seq analyses, such as those presented here, have unveiled a wealth of information about differentially expressed genes (DEGs) that are activated or repressed under drought conditions. These DEGs are often linked to critical pathways associated with stress signaling, hormone regulation, and osmotic adjustment [5]. Notably, recent advances in network analysis have allowed the identification of hub genes within co-expression modules, providing insights into key players governing mango's drought response.

The current study used Illumina sequencing to represent a comprehensive RNA-seq analysis of 36 mango samples, encompassing three distinct varieties subjected to varying degrees of drought stress. Our examination revealed the generation of clean reads spanning a range of 42,431,360 to 52,498,358; remarkably, a notable total of 12,752 differentially expressed genes (DEGs) emerged from this investigation. Intriguingly, an intriguing trend emerged from our findings, wherein downregulated genes exhibited a prevalence over upregulated genes in all pairwise comparisons, with exceptions observed in GLD *vs* GMD, JC *vs* GC, JHD *vs* GHD, TLD *vs* THD, and TMD *vs* THD.

To elucidate the genetic determinants underpinning mango's resilience to drought stress, the Weighted Gene Co-expression Network Analysis (WGCNA) emerged as a potent tool, identifying three pivotal genes at the forefront of drought tolerance. This novel approach enabled the identification of key genes that intricately regulate the complex machinery governing the plant's response to water scarcity. Through this analysis, three standout genes—*WRKY transcription factor 3* (LOC123206915), *polyamine oxidase 4* (LOC123226440), and *protein MEI2-like 1* (LOC123223059)—emerged as central players in mango's drought resistance mechanisms. WRKY transcription factors have long been recognized as master regulators of stress responses in plants. LOC123206915, a member of this family, takes the spotlight in our investigation. Previous studies have established the pivotal role of WRKY transcription factors in coordinating stress signaling pathways, orchestrating defense mechanisms, and modulating gene expression patterns to enhance plant resilience against environmental adversities [7]. This underscores the significance of LOC123206915 as a potential key regulator in mango's drought tolerance network. Polyamine oxidases are crucial players in the homeostasis of polyamine levels, and their involvement in stress adaptation has been widely documented [1]. Research has shown that polyamines mediate osmotic adjustment, scavenging reactive oxygen species and enhancing stress tolerance, thus underscoring the importance of LOC123226440 in mango defense against drought. Proteins often serve as effectors in mediating stress-induced responses. LOC123223059, encoding protein MEI2-like 1, is an intriguing candidate among the identified genes. Although its role in drought tolerance is less characterized, it is known that proteins like *MEI2-like 1* could play pivotal roles in signal transduction, regulatory pathways, and cellular responses to stress conditions [13]. Further exploration of this protein's functions may unravel novel facets of mango's drought resistance mechanism.

In a quest to comprehensively understand the multi-faceted response of Mango to varying levels of drought stress, namely light, moderate, and high, within the Guiqi (G), Jinhuang (J), and Tainong (T) varieties, we employed a widely targeted metabolome analysis. Notably, Pearson correlation analysis was leveraged to decipher the relationships between diverse groups based on the identified metabolites. The emergence of strong positive correlations, such as between GC and JC (r = 0.96), elucidates the coherent directional shift in metabolite profiles. This observation holds true for other pairs like *GHD vs JHD*, GLD *vs* JLD, indicating a consistent and correlated metabolic alteration among these conditions. This metabolite correlation matrix shows metabolomic harmony across distinct drought stress levels and varieties. As unveiled through robust correlations, the interplay of metabolite profiles amplifies our understanding of the intricate metabolic dynamics governing *M. indica's* response to drought stress. A staggering 1323 metabolites emerged as significant players in this intricate orchestra. Upon differential annotation, 990 metabolites stood out as key contributors, forming the basis for our subsequent investigations. These metabolites found their niche within 12 distinctive classes. Among these classes, amino acids and derivatives took center stage, amassing 333 metabolites. Not far behind, the phenolic acid class emerged as a prominent player, boasting 162 metabolites that could significantly influence the plant's defense strategies. The remaining classes, ranging from lipids and alkaloids to organic acids, nucleotide derivatives, lignans and coumarins, terpenoids, tannins, quinones, and others, contributed their unique essence to the tapestry. Together, these classes collectively painted a portrait of metabolic diversity in *M. indica* battle against drought stress, showcasing the plant's multi-faceted approach to adaptation.

### Integrating genes and metabolites

The interplay between genes and metabolites Figs. 1, 2, 3 and 5 have illegible texts, please provide replacement with clear texts, otherwise, we will proceed with the current figures.in mango's drought response reveals a fascinating narrative of adaptive mechanisms. Among the key genes identified, *WRKY transcription factor 3* (LOC123206915) emerges as a crucial orchestrator. This gene's involvement correlates with the accumulation of amino acids, including L-tryptophan, L-phenylalanine, and L-isoleucine. These amino acids serve as Osmo-protectants and contribute to stress signal transduction and metabolic reprogramming [7, 28]. *Polyamine oxidase 4* (LOC123226440) finds its significance in alignment with metabolites such as D-sphingosine and N2-Tryptophyllysine. D-sphingosine's role in modulating cellular responses to stress, including programmed cell death, aligns with polyamines' regulatory functions in drought adaptation [1]. N2-tryptophyllysine, potentially synthesized from L-tryptophan, might modulate protein functions under drought conditions, influencing plant stress responses [28].

The connection of protein *MEI2-like 1* (LOC123223059) with 2,5-Dimethyl pyrazine adds a layer of complexity. While the specific role of *MEI2-like 1* is less characterized, 2,5-Dimethyl pyrazine, known for its involvement in plant–microbe interactions and stress signaling, could point to a potential role of MEI2-like 1 in mediating stress-induced interplays [10].

In summary, the application of WGCNA has pinpointed three genes, *WRKY transcription factor 3*, *polyamine oxidase 4*, and *protein MEI2-like 1*, as crucial components of mango's defense against drought stress. As informed by previous research, the comprehensive understanding of these genes' functions validates our findings and opens doors to harnessing their potential for developing strategies to enhance drought tolerance in mango cultivation. The metabolite profiling expedition illuminated the richness and complexity of *M. indica* metabolic responses to drought stress. The diverse classes of metabolites showcased in this analysis enrich our understanding of the underlying mechanisms steering the plant's resilience and adaptation strategies. Collectively, the intricate cross-talk between genes and metabolites highlights and offers glimpses into the molecular strategies underlying mango's defense against drought stress.

## Conclusion

In conclusion, WRKY transcription factor 3, polyamine oxidase 4, and protein MEI2-like 1 were crucial for drought resistance in mango plants interacting with specific stress-associated metabolites. Collectively, this intricate cross-talk between genes and metabolites serves as an example, shedding light on the molecular strategies underpinning Mango's defense against the challenges of drought stress. These genetic and metabolic insights hold tremendous promise in driving future research and interventions aimed at fortifying mango cultivation against changing climates and ensuring sustained agricultural productivity. Future validation of these genes can provide a deep understanding and utilization in breeding drought-resistant mango varieties.

### Supplementary Information

**Supplementary Material 1.**

**Supplementary Material 2.**

## Data Availability

The raw data has been submitted to NCBI SRA under the project number: PRJNA1039530 (https://www.ncbi.nlm.nih.gov/sra/PRJNA1039530). Data will be released upon acceptance of this publication.
