# Supplementary material for: Integrating genes and metabolites: unraveling mango's drought resilience mechanisms
Source: BMC Plant Biol. 2024 Mar 23;24:208. doi: 10.1186/s12870-024-04908-w (PMC10960439; doi:10.1186/s12870-024-04908-w)
Supplement: Supplementary file 1 — Supplementary Material 1. [file 12870_2024_4908_MOESM1_ESM.docx]

| **Sample** | **Raw Reads** | **Clean Reads** | **Clean Base**  **(G)** | **Error Rate (%)** | **Q20**  **(%)** | **Q30**  **(%)** | **GC Content (%)** |
| --- | --- | --- | --- | --- | --- | --- | --- |
| TC-T-1 | 47602470 | 46284640 | 6.94 | 0.03 | 96.85 | 91.4 | 42.92 |
| TC-T-2 | 51623766 | 50512970 | 7.58 | 0.03 | 96.81 | 91.32 | 42.87 |
| TC-T-3 | 49098434 | 47739086 | 7.16 | 0.03 | 96.79 | 91.25 | 42.85 |
| JC-T-1 | 46576606 | 45538988 | 6.83 | 0.03 | 96.56 | 90.72 | 42.42 |
| JC-T-2 | 51193000 | 49872688 | 7.48 | 0.03 | 97.24 | 92.27 | 42.54 |
| JC-T-3 | 53837954 | 52498358 | 7.87 | 0.03 | 96.85 | 91.45 | 43.02 |
| GC-T-1 | 50675218 | 49807622 | 7.47 | 0.03 | 96.91 | 91.51 | 42.7 |
| GC-T-2 | 45683398 | 44885530 | 6.73 | 0.03 | 96.65 | 90.91 | 42.79 |
| GC-T-3 | 48962906 | 47679052 | 7.15 | 0.03 | 96.79 | 91.18 | 42.73 |
| TLD-T-1 | 50801862 | 49695540 | 7.45 | 0.03 | 96.78 | 91.23 | 42.09 |
| TLD-T-2 | 49009668 | 47877610 | 7.18 | 0.03 | 96.45 | 90.56 | 42.63 |
| TLD-T-3 | 48413360 | 47209280 | 7.08 | 0.03 | 96.9 | 91.48 | 42.63 |
| JLD-T-1 | 45958706 | 44785616 | 6.72 | 0.03 | 96.67 | 90.97 | 42.41 |
| JLD-T-2 | 47524726 | 46447794 | 6.97 | 0.03 | 96.59 | 90.81 | 42.41 |
| JLD-T-3 | 49413832 | 48457514 | 7.27 | 0.03 | 96.82 | 91.26 | 42.39 |
| GLD-T-1 | 48585900 | 47491574 | 7.12 | 0.03 | 97.27 | 92.26 | 42.68 |
| GLD-T-2 | 48582294 | 47420564 | 7.11 | 0.03 | 96.75 | 91.15 | 42.66 |
| GLD-T-3 | 47562660 | 46530610 | 6.98 | 0.03 | 96.82 | 91.29 | 42.84 |
| TMD-T-1 | 49181144 | 48155376 | 7.22 | 0.03 | 96.79 | 91.23 | 42.42 |
| TMD-T-2 | 46219920 | 45237802 | 6.79 | 0.03 | 96.75 | 91.13 | 42.41 |
| TMD-T-3 | 45571882 | 44371116 | 6.66 | 0.03 | 96.9 | 91.43 | 42.43 |
| JMD-T-1 | 48415684 | 46686840 | 7 | 0.03 | 96.98 | 91.63 | 42.38 |
| JMD-T-2 | 47395128 | 46537432 | 6.98 | 0.03 | 96.94 | 91.53 | 42.3 |
| JMD-T-3 | 43148308 | 42431360 | 6.36 | 0.03 | 96.63 | 90.85 | 42.02 |
| GMD-T-1 | 46805114 | 45564342 | 6.83 | 0.03 | 96.77 | 91.21 | 42.59 |
| GMD-T-2 | 48474508 | 47030418 | 7.05 | 0.03 | 97.1 | 91.87 | 42.34 |
| GMD-T-3 | 48303614 | 47365902 | 7.1 | 0.03 | 96.77 | 91.17 | 42.3 |
| THD-T-1 | 46803882 | 45553304 | 6.83 | 0.03 | 96.49 | 90.58 | 42.36 |
| THD-T-2 | 49809558 | 48801222 | 7.32 | 0.03 | 96.99 | 91.69 | 42.29 |
| THD-T-3 | 48601196 | 47186016 | 7.08 | 0.03 | 97.04 | 91.81 | 42.55 |
| JHD-T-1 | 44494322 | 43263452 | 6.49 | 0.03 | 96.8 | 91.22 | 42.6 |
| JHD-T-2 | 48461766 | 47147980 | 7.07 | 0.03 | 96.68 | 90.99 | 42.59 |
| JHD-T-3 | 46437208 | 44993760 | 6.75 | 0.03 | 96.51 | 90.6 | 42.54 |
| GHD-T-1 | 48094028 | 47189592 | 7.08 | 0.03 | 96.58 | 90.71 | 42.26 |
| GHD-T-2 | 47410854 | 46345450 | 6.95 | 0.03 | 96.71 | 91.02 | 42.38 |
| GHD-T-3 | 44971160 | 43767212 | 6.57 | 0.03 | 98.11 | 94.2 | 42.52 |

**Table S1**. Statistics of the transcriptome data


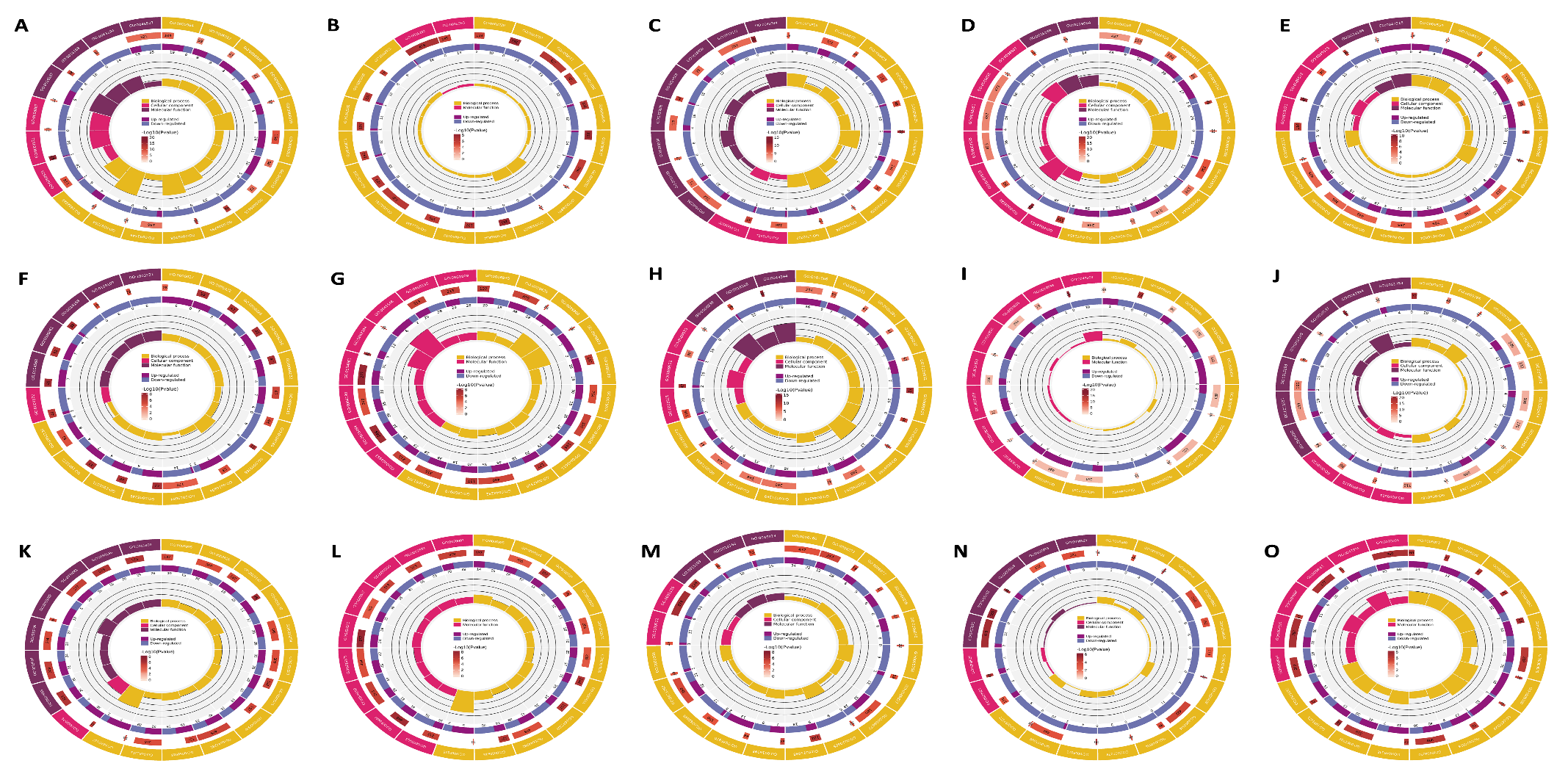


**Figure S1**. GO pathway enrichment of differentially expressed genes in three M. indica (Mango) germplasms under Low moderate and high level of drought stress.


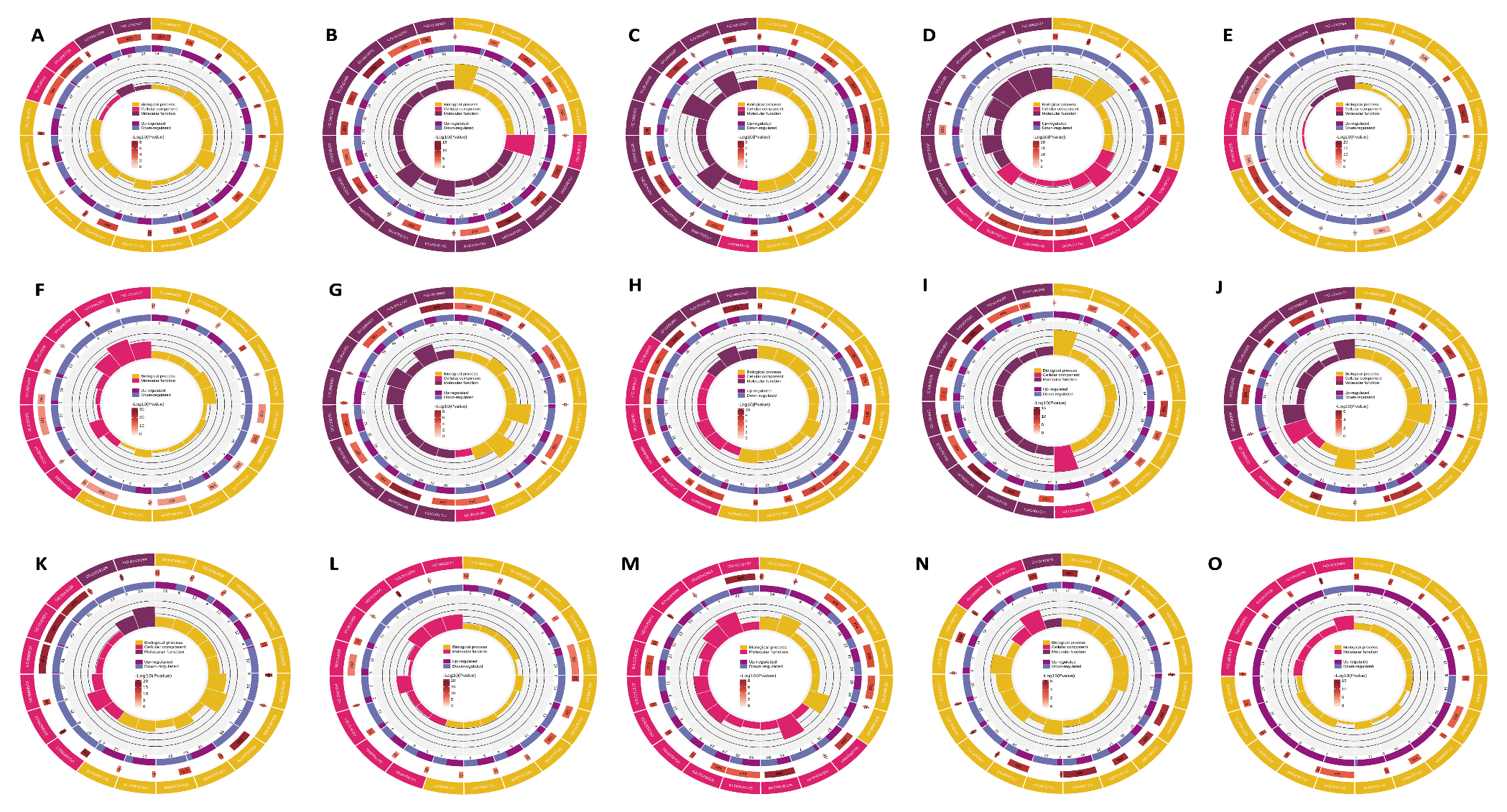


**Continuation of Figure S1.**


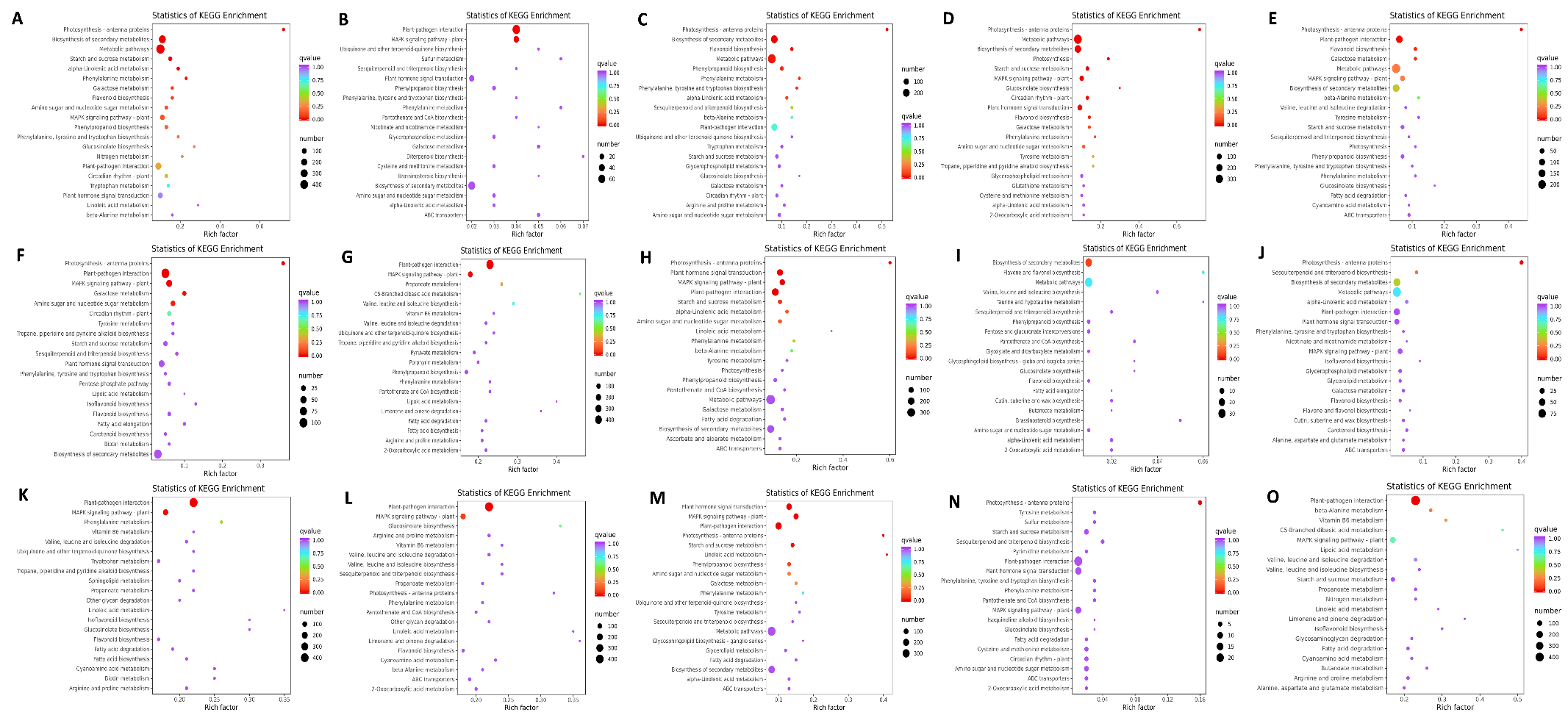


**Figure S2**. KEGG pathway enrichment of differentially expressed genes in three M. indica (Mango) germplasms under Low moderate and high level of drought stress


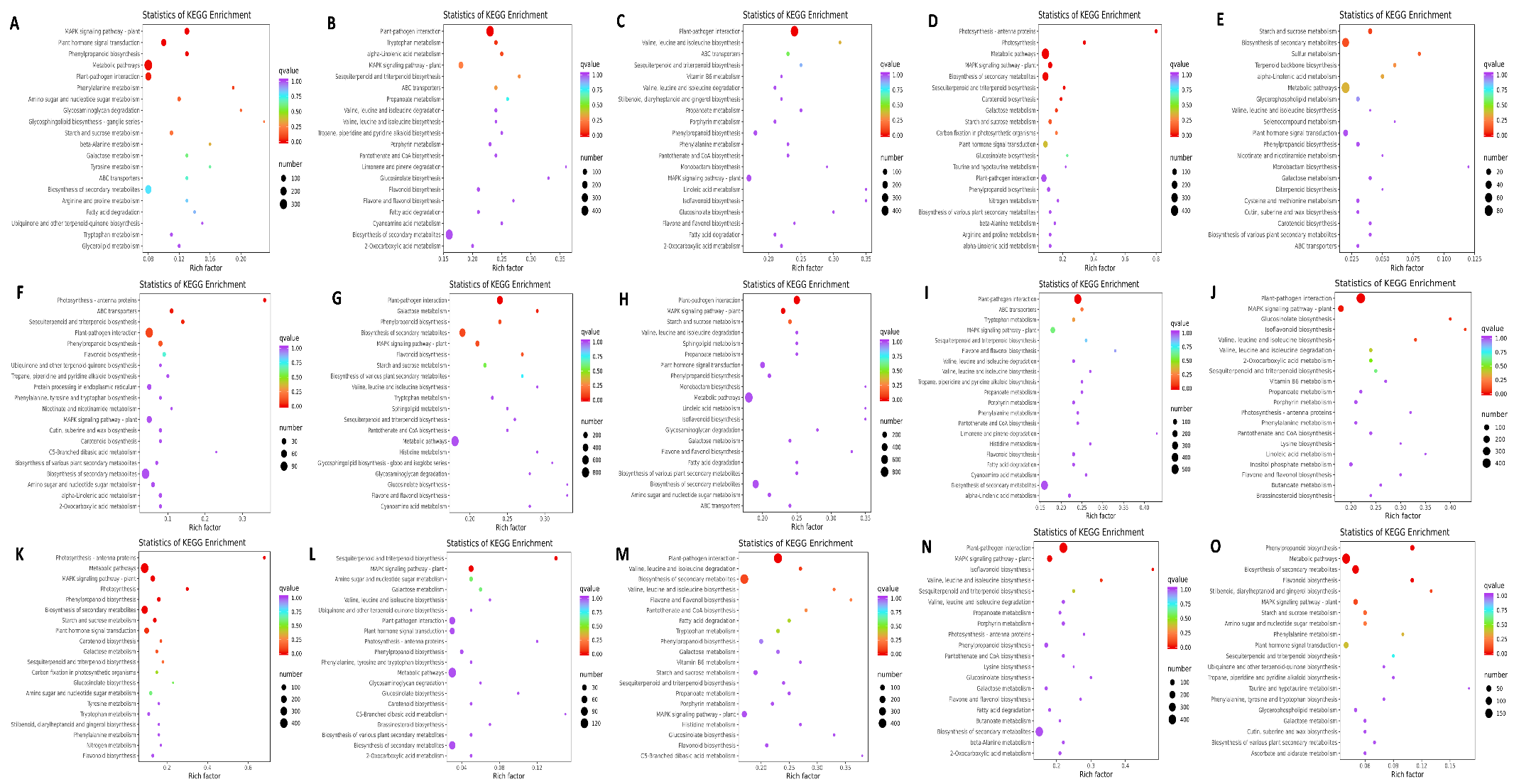


**Continuation of Figure S2.**


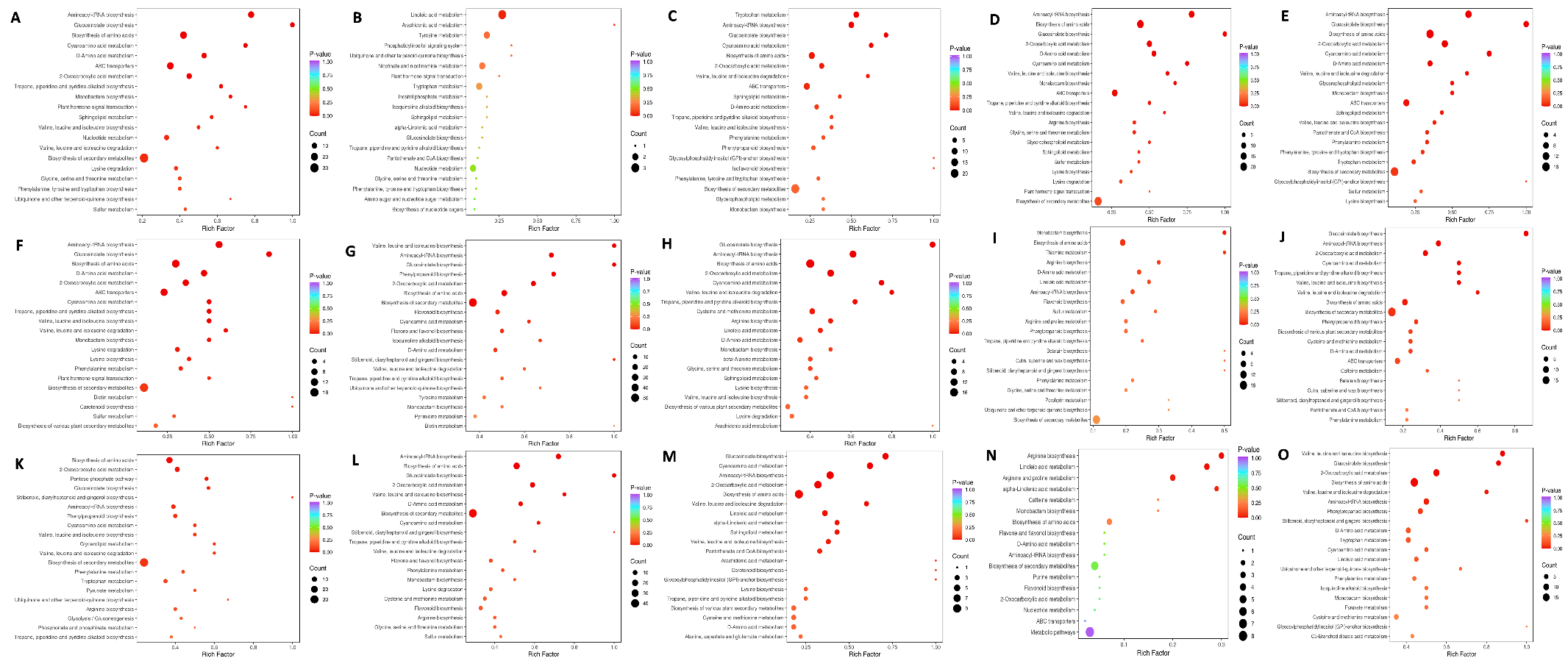


**Figure S3.** KEGG pathway enrichment of differentially annotated metabolites in three M. indica (Mango) germplasms under Low moderate and high level of drought stress.


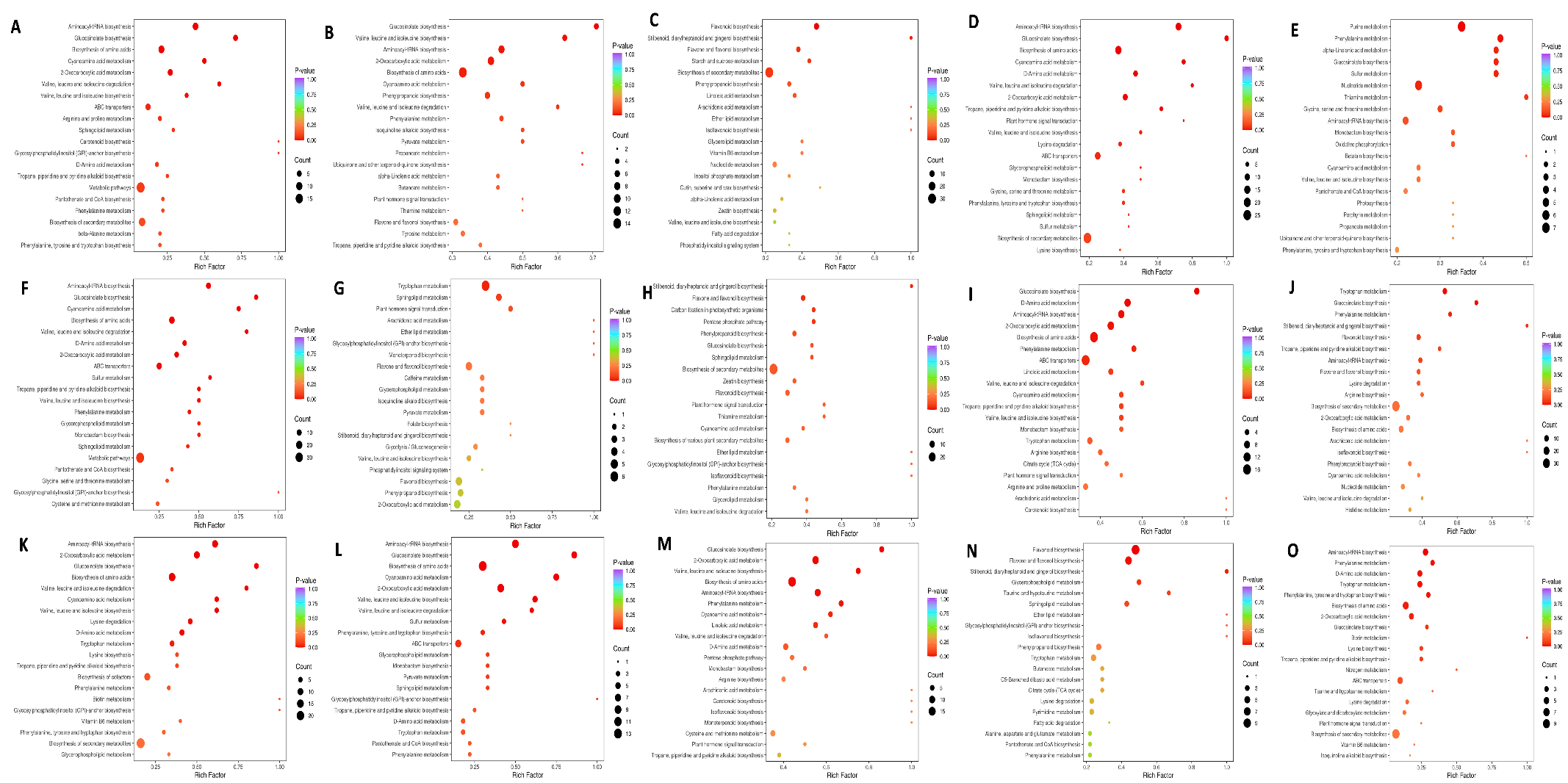


**Continuation of Figure S3.**
